# Clinical characteristics and drinking patterns among treatment‐seeking patients with alcohol use disorder in Japan from the ALPS‐J nationwide prospective cohort study

**DOI:** 10.1002/pcn5.70379

**Published:** 2026-08-07

**Authors:** Chie Nitta, Moemi Shibasaki, Yuko Urayama, Yosuke Yumoto, Hitoshi Maesato, Takeo Muto, Yuhei Amano, Yoshiki Koga, Tomomi Toyama, Ryuhei So, Kohei Kitagawa, Masayuki Ohishi, Masayuki Minami, Mitsuru Kimura, Sachio Matsushita

**Affiliations:** ^1^ National Hospital Organization, Kurihama Medical and Addiction Center Yokosuka Kanagawa Japan; ^2^ Institute of Medicine University of Tsukuba Tsukuba Ibaraki Japan; ^3^ National Hospital Organization, Ryukyu Hospital Kunigami Okinawa Japan; ^4^ Koyodai Hospital Kumamoto Japan; ^5^ Psychiatry Anzunokai Kakamigahara Hospital Kakamigahara Gifu Japan; ^6^ Okayama Psychiatric Medical Center Okayama Japan; ^7^ Department of Public Health Promotion and Human Behavior Kyoto University Graduate School of Medicine Kyoto Japan; ^8^ Ohishi Clinic Yokohama Kanagawa Japan; ^9^ Funabashi Kita Hospital Funabashi Chiba Japan

**Keywords:** alcohol use disorder, clinical characteristics, cohort, drinking patterns, treatment‐seeking patients

## Abstract

**Aim:**

While “controlled drinking” as a treatment goal for alcohol use disorder (AUD) has recently become accepted in Japan, knowledge regarding the clinical characteristics and drinking patterns of patients with AUD remains limited. This study aimed to describe the gender‐stratified baseline clinical characteristics and drinking patterns of treatment‐seeking patients with AUD using baseline data from the Alcohol Longitudinal Prognosis Study in Japan (ALPS‐J), a multicenter cohort study conducted at 15 facilities nationwide.

**Methods:**

The diagnosis and severity of AUD were assessed using the Structured Clinical Interview for DSM‐5 Research Version (SCID‐5‐RV). Depressive symptoms were assessed using the Patient Health Questionnaire‐9 (PHQ‐9). Sleep quality was assessed using the Japanese version of the Pittsburgh Sleep Quality Index (PSQI‐J). Drinking patterns were assessed using an original questionnaire.

**Results:**

A total of 354 participants were analyzed; 83.1% were male, the mean age was 51.9 ± 11.2 years, and 85.0% were inpatients. Overall, 80.8% met the diagnostic criteria for severe AUD. Treatment was started approximately 5 years after the onset of drinking problems. Comorbid depressive symptoms were observed in 44.7% of participants, and 74.3% reported poor sleep quality. The mean daily pure alcohol consumption was 110.4 g. The most commonly consumed type of alcoholic beverage was chu‐hai with 9% alcohol by volume, a high‐alcohol beverage commonly sold in Japan and often referred to as “strong‐style” chu‐hai.

**Conclusion:**

Patients with AUD treated in specialized addiction treatment facilities often present with severe and complex clinical profiles, highlighting the need for earlier engagement in care and integrated treatment approaches.

## INTRODUCTION

### Background for alcohol use disorder treatment in Japan

Alcohol use disorder (AUD) remains a major public health issue worldwide. In Japan, the prevalence of AUD is 5.7% in men and 1.4% in women (3.4% overall), which is lower than in Western and Southeast Asian countries.^1^ Although approximately 1.07 million individuals are estimated to have alcohol dependence,^2^ only around 75,000 receive treatment,^3^ indicating a substantial treatment gap. Historically, AUD treatment in Japan has relied heavily on inpatient psychiatric services. Following the 2013 Basic Act on Measures Against Alcohol‐related Health Harm, 233 specialized facilities were established nationwide. These services are covered by national insurance with minimal costs to patients at the point of service, and referral routes are diverse, including self‐referral, primary care providers, family members, employers, and government agencies. Historically, the treatment of alcohol dependence in Japan was developed primarily focusing on inpatient programs in psychiatric hospitals.

Despite these efforts, the proportion of individuals with AUD who receive specialized addiction treatment remains low. Stigma surrounding AUD and psychiatric services is a likely contributor to this treatment gap. The general population is reportedly reluctant to seek psychiatric care as a first‐line option, partly due to low mental‐health literacy.^4^ Such attitudes may deter individuals with AUD from presenting to psychiatric services and thus contribute to underdiagnosis. Moreover, drinking with colleagues or friends is a common form of social interaction in Japan, and societal attitudes toward alcohol consumption are relatively tolerant. This societal tolerance toward alcohol consumption is evident from the past‐year drinking prevalence rate of approximately 70% among adults (unpublished data, available on the National Center for Addiction Service Administration; NCASA's website). While heavy drinking is widely tolerated in Japan, being diagnosed with AUD carries a strong social stigma.^5^ In fact, another national survey found that 62.4% of respondents considered the development of AUD to be one's own responsibility.^6^ These sociocultural factors likely act as barriers to treatment access and contribute to the underdiagnosis of AUD.

Overall, the current sociocultural context indicates that the expansion of specialized facilities alone is insufficient to close the treatment gap, highlighting the need for policymakers and healthcare professionals to explore alternative approaches.

### Global shift in treatment goals and implications for Japan

Recently, a global trend has emerged toward more patient‐centered and flexible treatment goals that include not only abstinence but also “controlled drinking,” which permits alcohol use within agreed limits, thereby reducing psychological barriers to accessing treatment. Japan has followed this trend by revising its clinical guidelines for AUD in 2021 to incorporate more flexible treatment goals. The Japanese guidelines indicate that “controlled drinking” may be considered for patients with mild AUD without clear comorbid complications.^7^ However, this recommendation is largely based on clinical consensus, rather than robust empirical evidence from Japanese clinical samples. International studies have suggested that lower AUD severity, lower baseline alcohol consumption, fewer negative mood symptoms, and fewer heavy drinkers in the social network aid with achieving low‐risk drinking during treatment.^8^ Nevertheless, whether these findings apply to treatment‐seeking patients with AUD in Japan, where treatment access is often delayed, and specialized care is provided in more severe cases, remains unclear. Therefore, detailed baseline data are needed to identify patient profiles that may be suitable for reducing drinking goals.

### Limitations of previous research on the characteristics and drinking patterns of treatment‐seeking patients with alcohol use disorder

Internationally, large‐scale multicenter studies of patients with AUD or alcohol problems have included the COMBINE study, Project MATCH, and United Kingdom Alcohol Treatment Trial (UKATT).^9^, ^10^, ^11^ These randomized controlled trials provided important evidence of drinking outcomes using standardized assessments such as Form 90^12^ and reported detailed baseline information on drinking patterns, including the percentage of abstinent days and drinks per drinking day. However, as these studies recruited participants according to strict eligibility criteria for randomized trials, their samples may not have fully reflected patients seeking specialized addiction treatment in real‐world clinical settings. Moreover, as these studies were conducted several decades ago, their findings may not reflect the current clinical profiles, treatment backgrounds, or drinking patterns of treatment‐seeking patients with AUD. Recent naturalistic observational studies have examined treatment‐seeking patients with AUD in real‐world treatment settings, including single‐center treatment cohorts and samples from psychiatric hospitals.^13^, ^14^ However, these studies have generally focused on drinking trajectories, post‐treatment outcomes, comorbid psychiatric conditions, or withdrawal prognosis rather than providing a comprehensive multicenter baseline description of patients recruited from specialized addiction treatment facilities.

In Japan, several previous studies have reported selected clinical characteristics and drinking‐related variables in patients receiving specialized treatment for alcohol dependence or AUD.^15^, ^16^, ^17^, ^18^ However, most of these studies used single‐center or selected outpatient samples and focused on post‐treatment outcomes, the effects of smoking cessation, quality‐of‐life scale validation, and other specific treatment‐related factors. To the best of our knowledge, no previous study has provided comprehensive baseline data on the clinical characteristics and detailed drinking patterns of treatment‐seeking patients with AUD recruited from specialized addiction treatment institutions across multiple regions in Japan.

### Aims of this study

Comprehensive baseline data on the clinical characteristics and detailed drinking patterns of treatment‐seeking patients with AUD remain limited, particularly in real‐world specialized addiction treatment settings in Japan. Therefore, the present study aimed to describe the gender‐stratified baseline clinical characteristics and detailed drinking patterns of treatment‐seeking patients with AUD recruited from specialized addiction treatment institutions across multiple regions in Japan. These characteristics included AUD severity, treatment background, mental health status, sleep quality, alcohol consumption, drinking frequency, and preferred beverage type.

As the first report from the Alcohol Longitudinal Prognosis Study in Japan (ALPS‐J) cohort, this study provides baseline evidence for who accesses specialized AUD treatment in Japan.

## METHODS

### Study design

**Figure 1:**
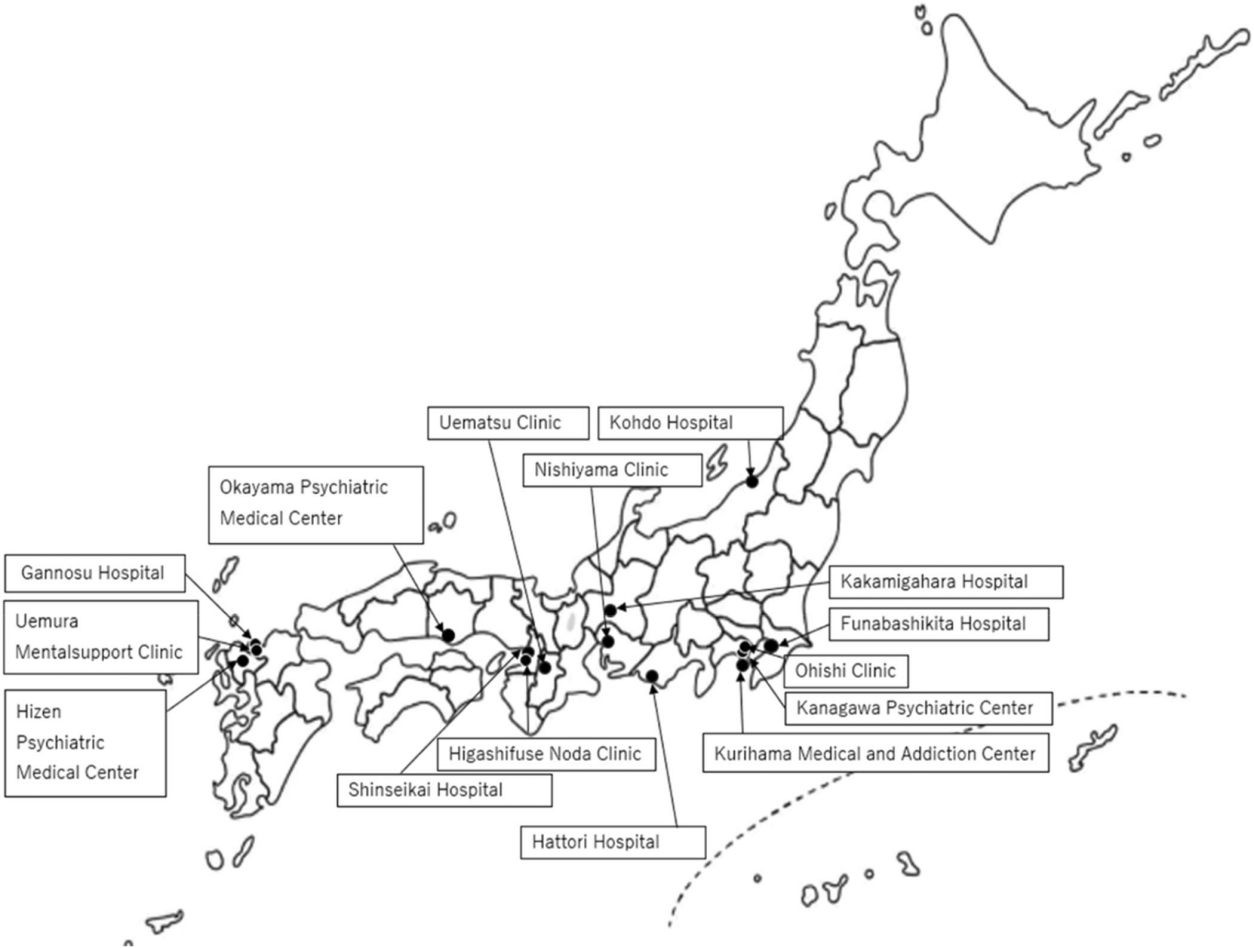
Study site locations in the Alcohol Longitudinal Prognosis Study in Japan (ALPS‐J) study.

The ALPS‐J is an ongoing naturalistic observational prospective cohort study that involves 15 medical institutions specializing in addiction treatment. Across Japan, facilities with a large number of new patients per year and qualifications for conducting clinical research were selected as collaborating institutions for this study. The facilities are listed at the end of this paper, and their locations are shown in Figure 1. Although the ALPS‐J includes longitudinal follow‐up assessments, in this study, we used only baseline data to describe the clinical characteristics and drinking patterns of treatment‐seeking patients with AUD.

### Participants

The participants were recruited from patients receiving standard care for AUD in Japan. In this study, the target clinical population was not the overall population of Japanese patients with AUD, but rather patients treated at specialized medical institutions providing structured treatment programs specifically for AUD. Specifically, standard treatment for AUD typically consists of a 2‐ to 3‐month inpatient program delivered in specialized psychiatric hospitals. This program includes psychoeducation, group cognitive behavioral therapy, occupational therapy, relapse prevention skills training, and pharmacotherapy. Outpatient treatment mainly consists of psychotherapy provided by psychiatrists, and day treatment programs are also available in some facilities. Outpatient care is offered either as aftercare following discharge from inpatient programs or as an initial treatment option for patients with relatively mild AUD. In Japan, approximately 13,000 individuals newly seek treatment for AUD at specialized addiction treatment institutions each year.

#### Inclusion criteria

The inclusion criteria for outpatients were as follows: age ≥ 18 years, a diagnosis of AUD, ability to provide written informed consent, clinical stability without severe acute symptoms, and willingness to comply with the treatment protocol and follow‐up assessments.

For inpatients, the same criteria applied, with the additional requirement that hospitalization for AUD treatment was expected to last more than 2 weeks.

#### Exclusion criteria

Exclusion criteria included severe physical illnesses that could interfere with study participation, acute suicidal ideation, severe aggression, and the need for immediate psychiatric intervention. Individuals with severe substance dependence who were non‐compliant with treatment recommendations were also excluded. Additional exclusion criteria were pregnancy or breastfeeding, inability to provide informed consent due to cognitive impairment, and participation in other clinical trials within the previous month. In addition, patients expected to be hospitalized for more than 90 days were excluded, as such prolonged admissions often reflect severe psychiatric comorbidities or the absence of appropriate discharge destinations, indicating that AUD treatment may not be the primary purpose of hospitalization. Individuals whose most recent discharge from the same facility occurred within the previous 6 months were also excluded, as frequent hospitalizations made it difficult to obtain reliable baseline information.

### Ethics

The participants provided written informed consent, and their personal information was recorded using a secure electronic data capture system. This study was approved by the ethical committee of the National Hospital Organization Kurihama Medical and Addiction Center, which served as the representative administration. It was also conducted in accordance with the Declaration of Helsinki—Ethical Principles for Medical Research Involving Human Subjects. In line with domestic ethical guidelines,^19^ ethical approval was obtained from the representative institution; however, some participating research sites also received formal approval from their respective medical ethics committees. Informed consent was obtained in writing after providing a full explanation of the study both orally and in writing by trained research staff, nurses, or physicians at each participating facility.

### Measures

The overall timeline and measurement instruments of this study are presented in Table S1.

#### Sociodemographic factors

Sociodemographic characteristics and information regarding sex, age, education, employment, economic status, living arrangements, marital status, cohabitation, smoking, and family history of addiction were obtained from medical records and semi‐structured interviews conducted by nurses trained as clinical research providers or specialized staff in AUD at collaborative facilities.

#### Assessment of AUD diagnosis and severity

AUD was diagnosed, and the severity of symptoms (mild, moderate, or severe) was assessed by an attending physician, typically a psychiatrist, using the Structured Clinical Interview for DSM‐5 Research Version (SCID‐5‐RV).^20^ In addition, sex differences in the prevalence of individual DSM‐5 diagnostic criteria were examined.

#### Mental health and sleep quality at the baseline

Depressive symptoms were assessed using the nine‐item Patient Health Questionnaire‐9 (PHQ‐9), scored 0–27. Each item reflects symptom frequency over the past 2 weeks (on a scale of 0–3). A score over 10 was used as the cut‐off, indicating clinically significant depressive symptoms.^21^ We used the open‐access Japanese version.^22^ Sleep quality was measured with the 19‐item Japanese version of the Pittsburgh Sleep Quality Index (PSQI‐J),^23^, ^24^ which is the validated translation of the original PSQI,^25^ yielding a global score of 0–21. Higher scores indicate poorer sleep quality. A global score over 5 was used as the cutoff for poor sleep quality. Details of the additional measures administered in the study are provided in Table S2.

#### Treatment history for AUD, physical comorbidities, and drinking history

Data on previous AUD treatment, including information on age at first visit for AUD treatment, treatment setting (inpatient or outpatient), pathways to specialized addiction treatment, history of substance use disorders other than alcohol, and participation in self‐help groups, were extracted from medical records and structured intake interview forms completed at the initial visit. In Japan, self‐help groups for AUD mainly include the Japan Sobriety Association (JSA), which emphasizes abstinence through community‐based support, and Alcoholics Anonymous. Physical comorbidities frequently associated with AUD were extracted from medical records and included liver dysfunction, hypertension, liver cirrhosis, diabetes mellitus, malignancy, pancreatitis, and a history of fracture. Drinking history variables included age at first drink, age at onset of habitual drinking, and age at onset of drinking problems. These variables were assessed by asking the following questions: “At what age did you first drink alcohol?,” “At what age did you begin drinking habitually (at least once a month)?,” and “At what age did you first feel that your drinking had become problematic?”

#### Alcohol drinking patterns and preferred type of alcoholic beverage during the 90 days prior to baseline

Data on drinking status were collected using a questionnaire developed for this study with reference to Form 90,^12^ a structured interview tool, to capture detailed information on alcohol drinking patterns. The participants were asked to report their drinking behavior during the 90 days prior to baseline. Survey items included their preferred type of alcoholic beverage and the unit in which it was consumed (number of cans or milliliters). Participants were asked about their consumption of six types of alcoholic beverages commonly consumed in Japan: chu‐hai, shochu, sake, beer, whisky, and wine. Chu‐hai is a Japan‐specific canned, carbonated soft drink–like alcoholic beverage, typically flavored with fruit juice or other additives to improve taste. It is inexpensive (approximately US$1 per can) and widely available at 24‐h convenience stores and drugstores throughout Japan. In addition, the participants were asked to report the total number of drinking days and the total number of heavy drinking days. Heavy drinking was defined as the consumption of six or more standard drinks (equivalent to ≥60 g of pure alcohol) on a single day, in accordance with the Japanese clinical standard. These items were used to calculate the amount of pure alcohol (g) the participants consumed during the period (see Table S2 for detailed questions). The amount of pure alcohol (g) was calculated as follows: pure alcohol (g) = volume consumed (mL) × alcohol concentration × 0.8 (specific gravity).

### Statistical analysis

Descriptive statistics were conducted on the participants' baseline demographic and clinical characteristics as well as their drinking status. These characteristics were presented stratified by sex. To compare males and females, chi‐square tests were used for categorical variables and *t*‐tests for continuous variables. For results with *p*‐values < 0.10, the effect sizes are reported in all tables. Furthermore, sex differences in alcohol consumption were examined, including the average daily pure alcohol consumption (g), total amount of pure alcohol consumption (g), and number of heavy drinking days (days). After confirming normality and homogeneity of variance, *t*‐tests were conducted for average daily pure alcohol consumption and total pure alcohol consumption, whereas the Mann–Whitney *U* test was used for heavy drinking days. Effect sizes have also been reported. All statistical analyses were conducted using spss software (version 28.0), with statistical significance defined as a two‐sided *p*‐value of <0.05.

## RESULTS

### Participant flow and enrollment

**Figure 2:**
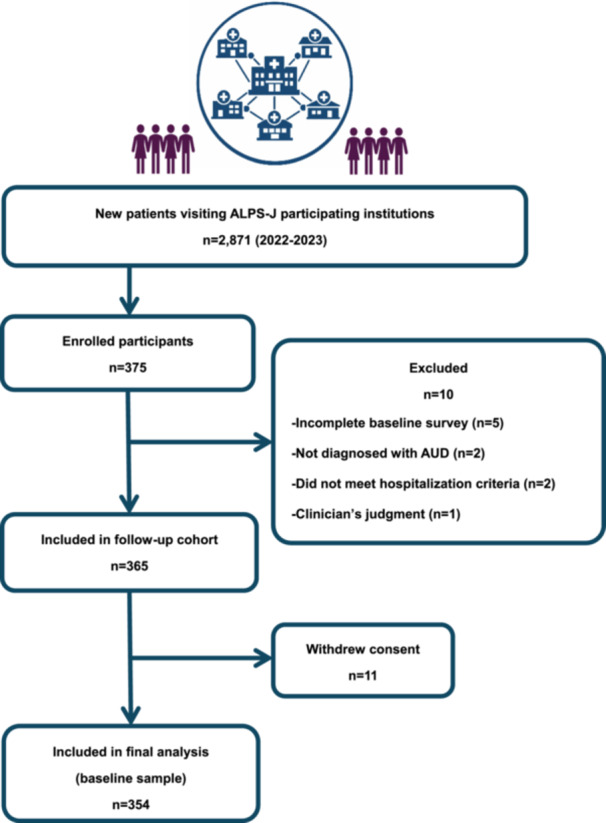
Alcohol Longitudinal Prognosis Study in Japan (ALPS‐J) participant flow. AUD, alcohol use disorder.

During the ALPS‐J recruitment period from April 2022 to March 2023, a total of 2871 new patients (2268 men and 603 women) visited the participating institutions, based on NCASA's original data (unpublished; available from the authors upon reasonable request). A total of 375 participants was enrolled across sites between April 2022 and March 2023. After those with incomplete baseline assessments or who were deemed ineligible (*n* = 10) were excluded, 365 participants were included in the follow‐up cohort. Finally, a total of 354 participants, excluding 11 who withdrew consent, was included in the final analysis (see Figure 2). The enrollment proportion (defined as the number of enrolled patients divided by the number of new patients during the recruitment period) across the 15 participating facilities ranged from 0.54% to 28.95% (mean, 13.45%; median, 9.86%; Table S3). This range indicates a substantial variation in enrollment proportion across sites. The sex distribution in the NCASA aggregate data was 79.0% men and 21.0% women, whereas that in the ALPS‐J analyzed sample was 83.1% men and 16.9% women.

### Sociodemographics of the participants

The sociodemographic, clinical, and mental health characteristics of the participants are presented in Table 1. Of the total sample, 294 were male (83.1%), and 60 were female (16.9%). The average age was 51.9 years (standard deviation [SD] = 11.2), with males being significantly older than females (53.0 vs. 46.8 years, *p* < 0.001, *d* = 0.561). Females were more likely to have attained higher education (*p* < 0.05, *φ* = 0.148). Males were more frequently employed as full‐time workers, while females were more likely to be part‐time workers (*p* < 0.01, *φ* = 0.443). Income from work was more commonly reported by males, whereas females more frequently received financial support from relatives (*p* < 0.05, *φ* = 0.121; *p* < 0.05, *φ* = 0.111). Having the status of current smoker was significantly more common among males (*p* < 0.001, *φ* = 0.228). Among those with a family history of substance use or behavioral addiction, alcohol‐related problems were the most common. Females were more likely than males to report a family history of gambling addiction (*p* < 0.05, *φ* = 0.110).

**Table 1:** Sociodemographic, clinical, and mental health characteristics of Alcohol Longitudinal Prognosis Study in Japan (ALPS‐J) participants.

	Total	Male	Female	*p* *
Sex	(*n* = 354)	(*n* = 294)	(*n* = 60)	
	100.0%	83.1%	16.9%	
Age	(*n* = 354)	(*n* = 294)	(*n* = 60)	
Mean ± SD (min–max)	51.9 ± 11.2 (21–80)	53.0 ± 10.7 (23‐80)	46.8 ± 12.4 (21‐77)	<0.001 (*d* = 0.561)
Education	(*n* = 353)	(*n* = 293)	(*n* = 60)	<0.05 (*φ* = 0.148)
<High school	12.7%	14.3%	5.0%	
High school	40.5%	42.0%	33.3%	
Some college or higher	46.7%	43.7%	61.7%	
Employment status	(*n* = 354)	(*n* = 294)	(*n* = 60)	<0.01 (*φ* = 0.443)
Self‐employed/full‐time worker	45.8%	50.3%	23.3%	
Part‐time worker	10.5%	9.2%	16.7%	
Housewife/househusband	3.4%	0.0%	20.0%	
Unemployed	36.2%	36.1%	36.7%	
Other (student)	4.2%	4.4%	3.3%	
Living expenses (multiple answers)	(*n* = 351)	(*n* = 292)	(*n* = 59)	
Income from work	53.8%	56.5%	40.7%	<0.05 (*φ* = 0.121)
Savings	8.3%	8.2%	8.5%	0.965
Pension	11.4%	12.0%	8.5%	0.426
Assistance from relatives	10.8%	9.2%	18.6%	<0.05 (*φ* = 0.111)
Social welfare	11.1%	11.6%	8.5%	0.466
Other	7.7%	6.2%	15.3%	<0.05 (*φ* = 0.125)
Living arrangements	(*n* = 354)	(*n* = 294)	(*n* = 60)	
Home	95.2%	94.6%	98.3%	0.41
Facility	3.1%	3.4%	1.7%	
Other	1.7%	2.0%	0.0%	
Cohabitants	(*n* = 354)	(*n* = 294)	(*n* = 60)	
Living alone	33.1%	35.0%	23.3%	0.079 (*φ* = 0.085)
Living with others	66.9%	65.0%	76.7%	
Marital status	(*n* = 352)	(*n* = 293)	(*n* = 59)	
Married	48.6%	47.4%	54.2%	0.810
Never married	25.0%	24.9%	25.4%	
Divorced	24.1%	25.3%	18.6%	
Widowed	2.0%	2.0%	1.7%	
Not answered	0.3%	0.3%	0.0%	
Smoking	(*n* = 350)	(*n* = 291)	(*n* = 59)	
Current smoker	56.0%	57.0%	50.8%	<0.001 (*φ* = 0.228)
Ex‐smoker	22.3%	25.1%	8.5%	
Never smoked	21.7%	17.9%	40.7%	
Family history of substance or behavioral addiction (multiple answers)				
	(*n* = 114)	(*n* = 93)	(*n* = 21)	
Alcohol	91.2%	92.5%	85.7%	0.908
llicit drug	0.9%	1.1%	0.0%	0.651
Gamble	18.4%	15.1%	33.3%	<0.05 (*φ* = 0.110)
Other	1.8%	2.2%	0.0%	0.522
Mental health and sleep quality				
PSQI	(*n* = 311)	(*n* = 259)	(*n* = 52)	
Good sleeper (≤5)	25.7%	25.5%	26.9%	0.828
Poor sleeper (>5)	74.3%	74.5%	73.1%	
PHQ‐9	(*n* = 338)	(*n* = 279)	(*n* = 59)	
Screen‐negative depression (<10)	55.3%	57.7%	44.1%	0.056 (*φ* = 0.104)
Screen‐positive depression (≥10)	44.7%	42.3%	55.9%	

*Note*: Data are presented as *n* (%) or mean ± standard deviation (range). For items allowing multiple responses, the denominator was the total number of respondents who answered the question, and the numerator was the number of respondents who selected each item.

Abbreviations: PHQ‐9, Patient Health Questionnaire‐9; PSQI, Pittsburgh Sleep Quality Index.

* *p*‐values for comparisons between males and females were calculated using *t*‐tests or chi‐square tests. Cohen's *d* and *φ* indicate effect sizes.

### Mental health and sleep quality at baseline

Depressive symptoms assessed by the PHQ‐9 showed an overall mean score of 9.27 (SD = 6.53; range 0–27), with mean scores of 8.99 (SD = 6.23) for males and 10.59 (SD = 7.70) for females. Approximately 45% of the total sample met the criteria for comorbid depressive symptoms. Sleep quality assessed by the PSQI showed an overall mean score of 9.18 (SD = 4.32; range 1–21), with mean scores of 9.18 (SD = 4.37) for males and 9.19 (SD = 4.12) for females, and more than 70% of the participants were classified as having poor sleep quality. No significant sex differences were observed for either PHQ‐9 or PSQI (*p* = 0.056, *φ* = 0.104) scores.

### AUD treatment history and drinking history

AUD treatment history and drinking history are summarized in Table 2. Most of the participants were referred for specialized addiction treatment by healthcare professionals. Nearly half of the participants had previous experience with AUD treatment. In addition, in 85.0% of the total sample, most participants received inpatient treatment.

**Table 2:** Treatment history for alcohol use disorder and drinking history of Alcohol Longitudinal Prognosis Study in Japan (ALPS‐J) participants.

	Total	Male	Female	*p* *
*Treatment setting*	(*n* = 354)	(*n* = 294)	(*n* = 60)	
Inpatient	85.0%	85.7%	81.7%	0.423
Outpatient	15.0%	14.3%	18.3%	
*Pathways to specialized addiction treatment* (multiple answers)				
	(*n* = 353)	(*n* = 293)	(*n* = 60)	
Referral by healthcare professionals	59.8%	58.7%	65.0%	0.350
Self‐referral	25.5%	25.9%	23.3%	0.683
Family‐initiated referral	27.2%	27.0%	28.3%	0.816
Other	4.2%	4.8%	1.7%	0.278
*History of physical comorbidities* (multiple answers)				
	(*n* = 354)	(*n* = 294)	(*n* = 60)	
Liver dysfunction	22.6%	21.4%	28.3%	0.244
Hypertension	20.1%	22.8%	6.7%	<0.001 (*φ* = 0.151)
Liver cirrhosis	11.9%	13.3%	5.0%	0.071
Diabetes mellitus	11.6%	13.6%	1.7%	<0.001 (*φ* = 0.140)
Malignancy	8.5%	8.5%	8.3%	0.966
Pancreatitis	8.2%	8.5%	6.7%	0.636
Fracture	6.5%	6.8%	5.0%	0.6
*Previous treatment for AUD*	(*n* = 354)	(*n* = 294)	(*n* = 60)	
Yes	51.4%	53.4%	41.7%	0.065
No	48.6%	46.6%	58.3%	
*Age at first visit to treatment for AUD*	(*n* = 178)	(*n* = 156)	(*n* = 22)	
Mean ± SD (min–max)	45.48 ± 11.09 (13–71)	45.92 ± 11.18 (13–71)	42.32 ± 10.12 (24–62)	0.154
*Participation in self‐help groups for AUD* (multiple answers)				
	(*n* = 349)	(*n* = 289)	(*n* = 60)	
Japan Sobriety Association (JSA)	15.8%	16.6%	11.7%	0.163
Alcoholics Anonymous (AA)	10.0%	10.4%	8.3%	
Other	3.7%	2.8%	8.3%	
Never participated	70.5%	70.2%	71.7%	
**Drinking history**				***p*** *
*Age at first alcohol use*	(*n* = 354)	(*n* = 294)	(*n* = 60)	
Mean ± SD (min–max)	17.7 ± 3.7 (1–30)	17.5 ± 3.7 (1–30)	18.4 ± 3.5 (6–27)	0.102
*Age at onset of habitual drinking*	(*n* = 350)	(*n* = 292)	(*n* = 58)	
Mean ± SD (min–max)	23.7 ± 7.9 (13–64)	23.2 ± 7.5 (13–64)	26.4 ± 9.2 (17–60)	<0.01 (*d* = 0.402)
*Age at onset of drinking problems*	(*n* = 331)	(*n* = 272)	(*n* = 59)	
Mean ± SD (min–max)	40.3 ± 13.6 (14–77)	40.8 ± 13.5 (14–72)	38.0 ± 13.7 (19–77)	0.150
*History of substance abuse other than alcohol* (multiple answers)				
	(*n* = 69)	(*n* = 59)	(*n* = 10)	
Methamphetamine	17.4%	16.9%	20.0%	0.979
Cannabis	27.5%	32.2%	0.0%	<0.05 (*φ* = 0.108)
Hallucinogens	8.7%	10.2%	0.0%	0.264
Organic solvents	17.4%	20.3%	0.0%	0.111
Prescription drugs	65.2%	62.7%	80.0%	0.874
Other	5.8%	6.8%	0.0%	1.000

*Note*: Data are presented as *n* (%) or mean ± standard deviation (range). For items allowing multiple responses, the denominator was the total number of respondents who answered the question, and the numerator was the number of respondents who selected each item.

Abbreviation: AUD, alcohol use disorder.

* *p*‐values for comparisons between males and females were calculated using *t*‐tests or chi‐square tests. Cohen's *d* and *φ* indicate effect sizes.

Compared with females, males were significantly more likely to have a history of hypertension and diabetes mellitus (hypertension: *p* < 0.001, *φ* = 0.151; diabetes mellitus: *p* < 0.001, *φ* = 0.140). However, because the female sample size was small and the effect sizes were small, these statistical findings should be regarded as tentative and interpreted with caution.

The mean age of first alcohol use and onset of habitual drinking was 17.7 and 23.7 years, respectively. Males started habitual drinking earlier than females (*p* < 0.01, *d* = 0.402). The mean age at onset of drinking problems was approximately 40 years, with no significant sex difference observed. In contrast, the mean age at first visit for AUD treatment was approximately 45 years, with no significant sex difference observed, indicating an average delay of about 5 years between the onset of drinking problems and initial contact with treatment services. Overall, a minority of participants reported substance use other than alcohol. A significant sex difference was observed for cannabis use (*p* < 0.05, *φ* = 0.108); however, this finding should be interpreted with caution because the number of females with a history of non‐alcohol substance use was small (*n* = 10). Participation in self‐help groups was reported by 29.5% of the participants, with the JSA being the most commonly attended. A majority had never participated in any self‐help group.

In addition, because our sample consisted mostly of inpatients and was unbalanced with respect to outpatients, we also compared their characteristics as a sensitivity analysis. As a result, significant differences were observed in employment status, previous AUD treatment history, drinking history, and PHQ‐9 scores: compared with outpatients, inpatients were more likely to be unemployed (*p* < 0.01, *φ* = 0.203), to have received prior AUD treatment (*p* < 0.001, *φ* = 0.194), and to have experienced depressive symptoms (*p* < 0.05, *φ* = 0.109). In contrast, outpatients reported earlier initiation of alcohol use and habitual drinking than inpatients (*p* < 0.01, *φ* = 0.683; *p* < 0.01, *φ* = 0.293) (see Table S4).

### DSM‐5 diagnostic criteria and severity of AUD

Based on the SCID‐5‐RV criteria, 80.8% of the participants were diagnosed with severe AUD. The most frequently met symptoms were Criterion 1 (using alcohol in larger amounts or over longer periods) and Criterion 2 (unsuccessful efforts to cut down). The least met was Criterion 8 (use in physically hazardous situations). There were no significant sex differences across the items of diagnostic criteria or in the distribution of AUD severity (see Table 3).

**Table 3:** Diagnostic and Statistical Manual of Mental Disorders, fifth edition (DSM‐5) diagnostic criteria and severity of alcohol use disorder in the Alcohol Longitudinal Prognosis Study in Japan (ALPS‐J) participants.

Diagnostic criteria for alcohol use disorder by DSM‐5		Threshold above			*p* *
		Total	Male	Female	
1	Alcohol is often taken in larger amounts or over a longer period than was intended	90.4%	89.5%	95.0%	0.341
2	There is a persistent desire or unsuccessful efforts to cut down or control alcohol use	89.0%	88.1%	93.3%	0.498
3	A great deal of time is spent in activities necessary to obtain alcohol, use alcohol, or recover from its effects	74.6%	74.5%	76.3%	0.563
4	Craving, or a strong desire or urge to use alcohol	76.6%	77.9%	71.2%	0.534
5	Recurrent alcohol use resulting in a failure to fulfill major role obligations at work, school, or home	59.3%	61.6%	50.8%	0.297
6	Continued alcohol use despite having persistent or recurrent social or interpersonal problems caused or exacerbated by the effects of alcohol	58.2%	57.2%	67.2%	0.089 (*φ* = 0.118)
7	Important social, occupational, or recreational activities are given up or reduced because of alcohol use	62.7%	63.9%	57.6%	0.551
8	Recurrent alcohol use in situations in which it is physically hazardous	29.1%	28.1%	36.8%	0.132
9	Alcohol use is continued despite knowledge of having a persistent or recurrent physical or psychological problem that is likely to have been caused or exacerbated by alcohol	80.5%	81.6%	78.0%	0.264
10	Tolerance	79.1%	79.2%	82.8%	0.577
11	Withdrawal	64.7%	66.8%	62.1%	0.743
	*Severity assessment*				
	Mild	6.8%	6.1%	10.0%	0.409
	Moderate	12.4%	11.9%	15.0%	
	Severe	80.8%	82.0%	75.0%	

*Note*: Data are presented as n (%).

Abbreviations: DSM‐5, diagnostic and statistical manual of mental disorders, fifth edition; SCID‐5‐RV, structured clinical interview for DSM‐5 research version.

* *p*‐values for comparisons between males and females were calculated using chi‐square test. *φ* values indicate effect sizes.

### Alcohol consumption and preferred beverage types at baseline

**Figure 3:**
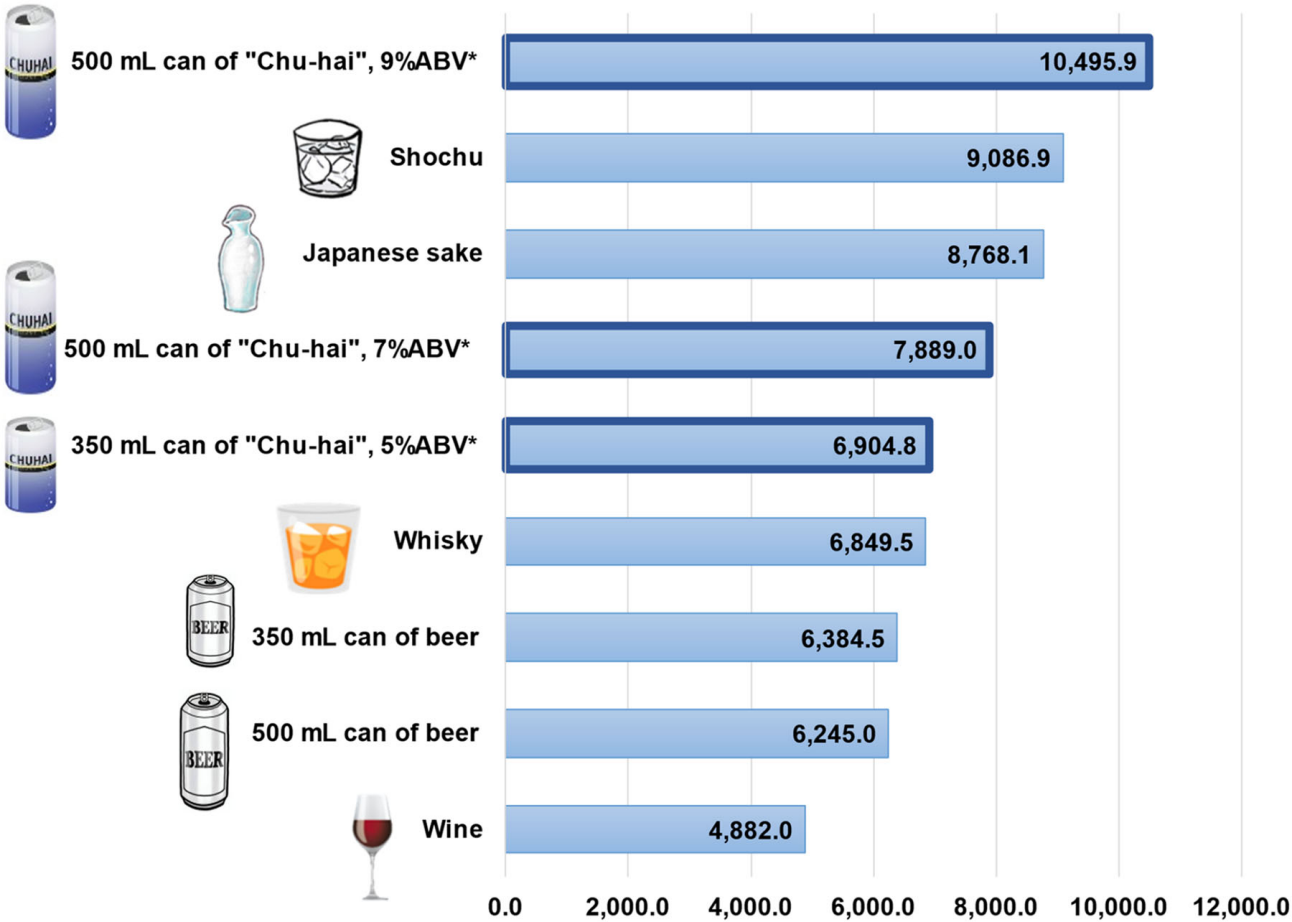
Mean pure alcohol intake by alcoholic beverage type among Alcohol Longitudinal Prognosis Study in Japan (ALPS‐J) participants during the 90 days prior to treatment initiation. *ABV, alcohol by volume. Values indicate the mean total amount of pure alcohol consumed by ALPS‐J participants during the 90 days prior to treatment initiation (g). “Chu‐hai” is a Japanese ready‐to‐drink alcoholic beverage. High‐alcohol varieties, such as those with 7% or 9% ABV, are often referred to as “strong‐style” chu‐hai.

As shown in Table 4, the mean average daily pure alcohol consumption among participants was 110.4 g (median: 100.0 g), and the mean total pure alcohol consumption over the 90 days prior to baseline was 8472.4 g (median: 7200.0 g). No significant sex differences were observed in either daily or cumulative alcohol consumption. Heavy drinking was prevalent, with a mean of 65.5 heavy drinking days and a median of 90 days over the 90‐day period, indicating almost daily heavy drinking among most participants. In addition, we calculated the mean amount of pure alcohol consumed by ALPS‐J participants during the 90 days prior to treatment initiation, stratified by type of alcoholic beverage (Figure 3). The highest mean pure alcohol consumption was observed for 500 mL cans of chu‐hai with 9% ABV, a ready‐to‐drink alcoholic beverage commonly available in Japan and generally referred to as “strong‐style” chu‐hai. These findings suggest that beverages with relatively high alcohol content contributed substantially to total alcohol consumption in this population.

**Table 4:** Alcohol consumption during the 90 days prior to baseline in the Alcohol Longitudinal Prognosis Study in Japan (ALPS‐J) participants.

Average daily pure alcohol consumption (g)	Total (*n* = 340)	Male (*n* = 284)	Female (*n* = 56)	*p* *	*d* ***
Mean ± SD (min–max) median	110.4 ± 70.6 (0.0–432.0) 100.0	113.2 ± 70.5 (0.0–432.0) 108.0	96.0 ± 70.2 (0.0–432.0) 77.8	0.091	0.244

**Total amounts of pure alcohol consumption (g)**	**Total (*n*** = **339)**	**Male (*n*** = **283)**	**Female (*n*** = **56)**	***p*** *	***d*** ***
Mean ± SD (min–max) median	8472.4 ± 6682.3 (0.0–38,880.0) 7200.0	8674.8 ± 6689.5 (0.0–38,880.0) 7344.0	7449.5 ± 6610.4 (0.0–38,880.0) 5850.0	0.210	0.184

**Number of heavy drinking days (days)**	**Total (*n*** = **304)**	**Male (*n*** = **258)**	**Female (*n*** = **46)**	***p*** **	
Mean ± SD (min–max) median	65.5 ± 31.1 (1.0–90.0) 90.0	65.8 ± 30.6 (1.0–90.0) 90.0	63.8 ± 34.2 (6.0–90.0) 90.0	0.987	

*Note*: All items regarding drinking status in the baseline survey referred to participants’ drinking habits during the 90 days before study entry; therefore, the results in this table represent participants’ drinking habits during the 90 days before treatment began. Data are presented as mean ± standard deviation, median, and range.

Abbreviation: SD, standard deviation.

* *p*‐values for comparisons between males and females were calculated using *t*‐tests. *d* values indicate effect sizes

** *p*‐values for comparisons between males and females were calculated using the Mann–Whitney *U* test.

*** Cohen's d indicates effect size.

## DISCUSSION

Our findings provide a comprehensive baseline profile of treatment‐seeking patients with AUD in Japan. This profile is characterized by a high prevalence of severe AUD, frequent heavy drinking, substantial psychiatric comorbidity, marked sex differences in sociodemographic and clinical features, and a preference for high‐alcohol‐content beverages, based on the first nationwide multi‐center cohort to systematically capture these clinical and behavioral characteristics.

### Clinical characteristics of treatment‐seeking patients with AUD

Analysis of the baseline characteristics of the 354 enrolled participants revealed that a substantial majority were male (83.1%), with a mean age of 51.9 years. Most of the participants also reported early initiation of alcohol use, a pattern strongly associated with more severe AUD presentations.^26^ The relatively high proportion of participants reporting a family history of alcohol or behavioral addiction in this study (approximately one‐third) suggests a potential genetic or environmental risk transmission. This rate falls between previous findings in studies of treatment‐seeking patients, which reported family histories of 27.4%^15^ and 44.9%,^16^ highlighting the continued clinical relevance of this factor.

In addition, most individuals did not seek treatment on their own initiative but were referred by healthcare professionals, such as primary care physicians and consultation services, highlighting the importance of systemic intervention in facilitating treatment entry. These findings are consistent with existing domestic literature. A prior study that investigated treatment‐seeking individuals with AUD in Japan^15^ reported a similar demographic distribution, including 85.2% male participation and a mean age of 53.9 years, along with comparable ages of initial alcohol use.

Moreover, our data showed that the mean age at the onset of the drinking problem was approximately 40 years. In contrast, the mean age at the first visit for AUD treatment was approximately 45 years, indicating an average delay of approximately 5 years between the onset of the drinking problem and initial contact with treatment services. Both the ALPS‐J study and a previous study^15^ highlighted a consistent pattern of delayed treatment‐seeking behavior, despite the early onset of problematic drinking. Internationally, although direct comparisons should be interpreted cautiously due to differences in study populations and definitions, the delay observed in our study appears to be within the range reported in previous studies. For example, previous research has indicated a median delay of 18 years in an Australian population‐based sample, a mean delay of 6.9 years in a Danish treatment sample, and a shorter lag of approximately 3 years in the US NESARC‐III study.^27^, ^28^, ^29^ In addition, in terms of the mean age of the patient population, the COMBINE study in the United States^9^ involved a younger cohort with a mean age of 44 years, whereas the mean age of the ALPS‐J participants was 51.9 years. This suggests that, despite similarly high levels of AUD severity, the population utilizing specialized treatment services in Japan may be older. These age differences may reflect variations in treatment accessibility, cultural perceptions of alcohol use, or the natural progression of AUD in the Japanese population. Collectively, these observations reinforce the critical need to promote earlier intervention and increase public awareness of AUD in Japan.

Assessment of depression and sleep quality revealed that 74.3% of the participants experienced insomnia symptoms, and 44.7% had comorbid depression. These findings are consistent with previous reviews reporting a high prevalence of insomnia and sleep disorders among individuals with AUD (36%–91% across studies).^30^ In addition, the frequent co‐occurrence of AUD and depression has been well established. Although an earlier systematic review highlighted that women with AUD are more likely than men to meet diagnostic criteria for major depressive disorder or dysthymia,^31^ no significant sex differences were observed in the present study. This discrepancy may be attributable to the relatively small number of female participants, resulting in limited statistical power to detect sex differences. That is, a type II error may have occurred due to sample size limitations. As the mean PHQ‐9 score was numerically higher among females, the absence of statistical significance should be interpreted cautiously. Depressive symptoms among female patients with AUD warrant further investigation with larger sample sizes. Given that both poor sleep quality and depressive symptoms are associated with poorer AUD outcomes,^30^, ^31^ clinicians treating this population should actively address these conditions through appropriate intervention and symptom management. Participants in the ALPS‐J study frequently presented with gastrointestinal diseases such as liver dysfunction, liver cirrhosis, and pancreatitis, diabetes mellitus, and malignancy. This may indicate that treatment is often sought only after the physical condition of the patient has substantially deteriorated. These findings highlight the importance of early intervention in collaboration with internal medicine departments. General medical settings may offer opportunities for the earlier identification of problematic alcohol use before substantial physical deterioration occurs.

### Drinking patterns and preferred beverage types of treatment‐seeking patients with AUD

At baseline, the participants demonstrated a pattern of frequent and high‐volume alcohol consumption. The median daily consumption of pure alcohol was 100.0 g, and the median number of drinking days in the past 90 days was 90, suggesting consistent daily heavy drinking. These values are characteristic of severe AUD. The types of alcoholic beverages consumed played a substantial role in total alcohol consumption. Strong chu‐hai products—a low‐cost, high‐alcohol (9%), flavored ready‐to‐drink beverage that effectively masks the taste of alcohol, thereby facilitating rapid intoxication—were the most commonly consumed. This preference is distinctive in comparison with the wine and beer preferences often reported in Western studies. These beverages have raised concerns among public health experts due to their potential role in exacerbating problematic drinking behaviors. Their affordability, widespread availability, and aggressive marketing strategies in Japan^32^ contribute to their frequent consumption. Previous studies have shown the link between strong chu‐hai consumption and hazardous drinking patterns, owing to the palatability and low cost of this type of drink.^33^ Shochu, another high‐alcohol (approximately 25%) beverage, was also widely consumed. This drinking pattern contrasts with those observed internationally, such as in France, where individuals with AUD typically prefer wine and beer.^34^ These differences underscore the influence of cultural and economic factors on drinking behaviors. In Japan, the widespread popularity of strong chu‐hai presents a distinct public health challenge.

### Comparison of clinical characteristics and drinking status between inpatients and outpatients

The present study included a greater proportion of inpatients than outpatients. Therefore, we compared sociodemographic characteristics and drinking‐related variables between the two groups. Significant differences were observed in employment status, history of AUD treatment, and drinking history. Specifically, inpatients were more likely to be unemployed, whereas outpatients were more likely to be employed full‐time. Inpatients were also more likely to have a history of AUD treatment. In contrast, outpatients reported an earlier age of first alcohol use and earlier onset of habitual drinking. However, no significant differences were observed between groups in terms of baseline alcohol consumption or AUD severity (Tables S4 and S5). As patients in Japan often cycle between inpatient and outpatient care, there were no substantial differences in drinking status or severity between inpatients and outpatients. Nevertheless, as the present sample included a large proportion of inpatients and many patients with physical comorbidities, it may not represent the primary target population for “controlled drinking”. However, reducing alcohol consumption may still have clinical value as a harm‐reduction approach for patients who are not immediately ready to accept abstinence.

### Strengths and limitations

This study had several limitations. First, the sample may have been subject to selection bias, which limits its generalizability. Although recruitment was conducted across 15 specialized addiction treatment institutions nationwide, no facilities in the Tohoku region were included, resulting in uneven regional representation. Therefore, the results should be interpreted with caution, and future studies should include specialized facilities in all regions of Japan. Moreover, the enrollment proportion varied substantially across facilities (0.54%–28.95%), likely reflecting the differences in institutional size and available research resources. Such variations may have affected the representativeness of the sample and generalizability of the findings. In particular, patients who were more motivated to engage in treatment or willing to participate in research may have been overrepresented. This bias may also have led to an overrepresentation of relatively severe AUD cases, potentially resulting in an overestimation of baseline alcohol consumption, thereby distorting the findings.

Second, the male‐to‐female ratio in our sample was approximately 5:1, with relatively few women. This ratio may reflect the lower prevalence and poorer treatment‐seeking rates of AUD among women in Japan (approximately one‐sixth that of men),^35^ rather than a recruitment bias. Nevertheless, women with AUD face unique barriers to treatment and often present with greater clinical and psychosocial burdens than men.^36^ Therefore, future studies should include larger sample sizes of female patients.

Finally, this study has several measurement‐related limitations. Most indicators, including alcohol consumption, were based on self‐reports and may therefore be subject to recall and social desirability biases.

Despite these limitations, ALPS‐J is Japan's first nationwide, multicenter cohort study involving addiction treatment facilities across the country. A major strength of this study is the use of continuous outcome measures to capture baseline drinking patterns, allowing for a detailed characterization of patients' drinking patterns. In addition, the inclusion of treatment‐seeking patients from diverse clinical settings reflects real‐world addiction care. This approach may also enhance the relevance of the findings for international research on AUD treatment in diverse sociocultural contexts.

## CONCLUSION

This study describes the sex‐stratified baseline clinical, behavioral, and mental health characteristics of treatment‐seeking patients with AUD in Japan. Patients with AUD receiving care at specialized addiction treatment facilities often present with severe and complex clinical profiles, highlighting the need for earlier engagement in care and integrated treatment approaches. In Japan and internationally, these findings provide an essential baseline for interpreting the characteristics of patients with AUD in societies where alcohol use is culturally accepted and access to treatment is delayed by barriers to care.

## AUTHOR CONTRIBUTIONS

Chie Nitta was the lead researcher and played a central role in study conceptualization, supervision, and manuscript preparation. She contributed to conceptualization, methodology, project administration, data curation, and writing original drafts. Moemi Shibasaki contributed to conceptualization, methodology, project administration, and formal analysis. Yuko Urayama was involved in data curation, visualization, writing original drafts, and formal analysis. Yosuke Yumoto, Hitoshi Maesato, Takeo Muto, and Yuhei Amano contributed to the investigation and provided clinical resources for data collection. Yoshiki Koga contributed to project administration. Masayuki Ohishi, Masayuki Minami, and Mitsuru Kimura provided essential resources. Tomomi Toyama, Ryuhei So, and Kohei Kitagawa contributed to conceptualization, methodology, and manuscript review. Sachio Matsushita was the principal investigator and contributed to supervision and funding acquisition. All the authors contributed to the interpretation of findings, critically reviewed the manuscript, and approved the final version for submission.

## CONFLICT OF INTEREST STATEMENT

R.S. reports personal fees from CureApp, Inc. during the conduct of the study; grants from the Osake‐no‐Kagaku Foundation, Mental Health Okamoto Memorial Foundation, and Kobayashi Magobe Memorial Medical Foundation; and personal fees from Otsuka Pharmaceutical Co., Ltd., Nippon Shinyaku Co., Ltd., Sumitomo Pharma Co., Ltd., and Takeda Pharmaceutical Co., Ltd., outside the submitted work. In addition, R.S. has pending patents: JP2022049590A, US20220084673A1, JP2022178215A, JP2022070086, and JP2023074128A.

## ETHICS APPROVAL STATEMENT

This study was approved by the ethical committee of the National Hospital Organization Kurihama Medical and Addiction Center (Approval No. 403), which served as the representative administration. It was also conducted in accordance with the Declaration of Helsinki—Ethical Principles for Medical Research Involving Human Subjects.

## PATIENT CONSENT STATEMENT

The participants provided written informed consent, and their personal information was recorded using a secure electronic data capture system.

## CLINICAL TRIAL REGISTRATION

N/A.

## Supporting information

Supporting File 1.

## Data Availability

The data that support the findings of this study are available from the first author upon reasonable request. The data are not publicly available due to privacy and ethical restrictions.
