# Supplementary material for: Clinical characteristics and drinking patterns among treatment‐seeking patients with alcohol use disorder in Japan from the ALPS‐J nationwide prospective cohort study
Source: PCN Rep. 2026 Aug 7;5(3):e70379. doi: 10.1002/pcn5.70379 (PMC13449294; doi:10.1002/pcn5.70379)
Supplement: Supplementary file 1 [file PCN5-5-e70379-s001.docx]

**S1 Table: Survey timeline and measures**

| **Measure** | **Survey timeline** | | **Follow up periods** | | | |
| --- | --- | --- | --- | --- | --- | --- |
|  | **Baseline** | **Discharge** | **3 months** | **6 months** | **12 months** | **Every**  **4 weeks** |
| ***Sociodemographic variables*** | | | | | | |
| Sex, Age | ✓ |  |  |  |  |  |
| Education, Employment status, Living expenses, | ✓ |  |  |  |  |  |
| Residence type, Cohabitants, Marital status | ✓ |  |  |  |  |  |
| Smoking status, Family history of substance or behavioral addiction | ✓ |  |  |  |  |  |
| ***History of alcohol problems*** | | | | | | |
| Access pathway to current treatment facilities | ✓ |  |  |  |  |  |
| Prior-treated experiences of alcohol problem | ✓ |  |  |  |  |  |
| Drinking history (Age of first drinking, habitual drinking, onset age of drinking problem) | ✓ |  |  |  |  |  |
| History of substance abuse other than alcohol | ✓ |  |  |  |  |  |
| ***Information for AUD diagnosis and other clinical conditions*** | | | | | | |
| SCID-5-RV (diagnosis for AUD) | ✓ |  |  |  |  |  |
| CIWA-Ar (Alcohol withdrawal) | ✓ |  |  |  |  |  |
| Liver function tests (γ-GTP, ALT, AST) | ✓ | ✓ | ● | ● | ● |  |
| Medical history  (mental illness/physical illness) | ✓ |  |  |  |  |  |
| Alcohol Dependence Scale (ADS) | ✓ |  |  |  |  |  |
| ***Alcohol treatment status*** |  |  |  |  |  |  |
| Participant’s drinking goals | ✓ | ✓ | ✓ | ✓ | ✓ |  |
| Participation in self-help groups | ✓ | ✓ | ✓ | ✓ | ✓ |  |
| Types of medication for AUD | ✓ | ✓ | ✓ | ✓ | ✓ |  |
| ***Treatment status during follow-up periods*** | | | | | | |
| Current residential status (facility/home) | ✓ | ✓ | ✓ |  |  |  |
| Types of current AUD treatment  (inpatient/outpatient/ day treatment /personal counseling) | ✓ | ✓ | ✓ |  |  |  |
| Current adherence of pharmacotherapy for AUD | ✓ | ✓ | ✓ |  |  |  |
| ***Drinking status **** |  |  |  |  |  |  |
| Total number of drinking days | ✓ |  |  |  |  | ✓ |
| Average daily pure alcohol consumption (g) | ✓ |  |  |  |  | ✓ |
| Total number of heavy drinking days | ✓ |  |  |  |  | ✓ |
| Total amounts of pure alcohol consumption for 90 days or 28 days (g) | ✓ |  |  |  |  | ✓ |
| Total number of days until drinking restarted |  |  |  |  |  | ✓ |
| Total number of days until heavy drinking restarted |  |  |  |  |  | ✓ |
| Type of consumed alcohol beverages | ✓ |  |  |  |  | ✓ |
| ***Mental health and psycho-social variables*** | | | | | | |
| Depression (PHQ9) | ✓ | ✓ | ✓ | ✓ | ✓ |  |
| Sleep status (Pittsburgh sleep quality index) | ✓ | ✓ | ✓ | ✓ | ✓ |  |
| QOL (SF12v2) | ✓ |  |  | ✓ | ✓ |  |
| Social Support Scale | ✓ |  | ✓ | ✓ | ✓ |  |
| Self-esteem (Rosenberg's Self-Esteem Scale) | ✓ | ✓ |  | ✓ | ✓ |  |

**Note**: *Drinking status; in the baseline survey, information about drinking status for the 90 days prior to study enrollment was collected, in the follow-up survey, those was collected every 4 weeks (28 days).

“●” indicates any variables measured via the retrospective medical record review from collaborating facilities after 1-year from baseline.

All of information regarding drinking status based on the participant’s self-report.

**Abbreviations**: SCID-5-RV: Structured Clinical Interview for DSM-5, Research Version, CIWA-Ar: Clinical Institute Withdrawal Assessment for Alcohol, Revised, γ-GTP: Gamma-Glutamyl Transpeptidase, ALT: Alanine Aminotransferase, AST: Aspartate Aminotransferase, AUD: Alcohol Use Disorder, ADS: Alcohol Dependence Scale, SF-12v2: Short Form Health Survey version 2, PHQ-9: Patient Health Questionnaire-9.

**S2 Table: The items of drinking status survey**

|  | **Question Statement** | **Answer** |
| --- | --- | --- |
| Q1 | What was the last date you drank any alcoholic beverage | Year ____ / Month____/ Day ___ |
| Q2 | During this period, how frequently did you consume even a single sip of an alcoholic beverage? Choose one  If you selected 2–11, go to Q4.  If you selected 1, go to Q3 | 1. Less than once/month  2. 2 days/month  3. 3 days/month  4. 4 days /month  5. 1 day/week  6. 2 days/week  7. 3 days/week  8. 4 days/week  9. 5 days/week  10. 6 days/week  11. Every day |
| Q3 | If you selected “1. Less than once/month” in Q2, how many days in total did you drink during this period? | __ days |
| Q4 | What type of alcoholic beverage did you drink most frequently during this period?  Even if you drink a combination of several types of alcoholic beverages, please choose just one and mark it with a circle in the illustration. | Write your answer in the table below. |
| Q5 | During this period, how much of the alcoholic beverage selected in Q4 did you consume on average per day?  Please write the amount you drank in the blank space.  If you diluted the beverage (e.g., with water or soda), please report the amount of the undiluted alcohol. | Write your answer in the table below. |

**S2 Table Continued**

**The items of drinking status survey**

| **Beer** | |
| --- | --- |
|  |  |
| Alcohol by volume, 5% | |
| 350mL/can | 500ml/can |
| can(s) | can(s) |

| **Chu-hai** | | | | | |
| --- | --- | --- | --- | --- | --- |
|  | | |  | | |
| 350ml/can | | | 500mL/can | | |
| Alcohol by volume category | | | Alcohol by volume category | | |
| 5% | 7% | 9% | 5% | 7% | 9% |
| can(s) | can(s) | can(s) | can(s) | can(s) | can(s) |

| **Shochu** | **Whisky** | **Wine** | **Japanese Sake** |
| --- | --- | --- | --- |
|   Alcohol by volume, 25% |  |  |  |
| １ ”gou” *  (180 mL, neat) | single (30 mL, neat)  doble (60 mL, neat) | One glass  (120 mL, neat) | １ ”gou”  (180 mL, neat) |
| mL | mL | mL | mL |

**S2 Table Continued**

**The items of drinking status survey**

|  | **Question Statement** | **Answer** |
| --- | --- | --- |
| Q6 | Next, we would like to ask about days when you consumed a large amount of alcohol.  During this period, were there any days when you drank more than the amount shown on the last page of the alcoholic beverage selected in Q4?  If you selected “1. Yes,” proceed to Q7.  If you selected “2. No,” the survey ends here. | 1. Yes  2. No |
| Q7 | During this period, on what date did you first consume a large amount of the alcoholic beverage selected in Q4? | ___ Month ___ Day |
| Q8 | During this period, how often did you have heavy drinking days with the alcoholic beverage selected in Q4? Please choose the one option that best applies to your situation.  If you selected “1,” proceed to Q9.  If you selected options 2 through 11, the survey ends here. | 1. Less than once/month  2. 2 days/month  3. 3 days/month  4. 4 days /month  5. 1 day/week  6. 2 days/week  7. 3 days/week  8. 4 days/week  9. 5 days/week  10. 6 days/week  11. Every day |
| Q9 | Only for those who selected “1. Less than once a month” in Q8.  How many heavy drinking days did you have in total during this period? | ____ days |

**Note:** Participants were asked about their alcohol use in the 90 days prior to their last drink date reported in "Q1"

1 “gou” (合) is a traditional Japanese unit of volume used for sake. It’s equivalent to approximately 180 milliliters, or about 6 fluid ounces."

Abbreviation: ALPS-J, Alcohol Longitudinal Prognosis Study in Japan

**S3 Table: Enrollment proportion by participating sites in the ALPS-J**

| **Site** | **Number of new patients**  **(n)** | **Number of enrolled participants (n)** | **Enrollment proportion (%)** |
| --- | --- | --- | --- |
| A | 101 | 26 | 25.74% |
| B | 294 | 29 | 9.86% |
| C | 176 | 31 | 17.61% |
| D | 234 | 52 | 22.22% |
| E | 82 | 5 | 6.10% |
| F | 214 | 27 | 12.62% |
| G | 207 | 11 | 5.31% |
| H | 123 | 24 | 19.51% |
| I | 551 | 3 | 0.54% |
| J | 83 | 6 | 7.23% |
| K | 360 | 91 | 25.28% |
| L | 57 | 3 | 5.26% |
| M | 76 | 22 | 28.95% |
| N | 151 | 14 | 9.27% |
| O | 162 | 10 | 6.17% |
| Total | 2871 | 354 | 12.33% |
| Mean (SD) |  |  | 13.45% |
| Median |  |  | 9.86% |
| Range |  |  | 0.54% - 28.95% |

Note: Note: Enrollment proportion was defined as the number of enrolled participants divided by the number of new patients during the recruitment period at each participating site.

Participating sites were masked and labeled alphabetically (Sites A–O) to avoid identification of individual institutions.

**S4 Table:**

**Comparison of sociodemographic, clinical, mental health, and drinking-related characteristics between inpatients and outpatients in the ALPS-J participants**

|  | **Total** | **Inpatients** | **Outpatient** | ***p**** |
| --- | --- | --- | --- | --- |
| ***Sex*** | **(n = 354)** | **(n = 301)** | **(n = 53)** |  |
| Male | 83.1% | 83.7% | 79.2% | 0.423 |
| Female | 16.9% | 16.3% | 20.8% |  |
| ***Age*** | **(n = 354)** | **(n = 301)** | **(n = 53)** |  |
| Mean ± SD (min-max） | 51.9±11.2 (21-80) | 52.1±11.0 | 50.8±12.5 | 0.430 |
| ***Education*** | **(n = 353)** | **(n = 300)** | **(n = 53)** |  |
| < High school | 12.7% | 12.3% | 15.1% | 0.252 |
| High school | 40.5% | 42.3% | 30.2% |  |
| Some college or higher | 46.7% | 45.3% | 54.7% |  |
| ***Employment status*** | **(n = 354)** | **(n = 301)** | **(n = 53)** |  |
| Self-employed/Full time worker | 45.8% | 43.2% | 60.4% | <.01  (φ = 0.203) |
| Part-time worker | 10.5% | 11.0% | 7.5% |  |
| Housewife/househusband | 3.4% | 2.7% | 7.5% |  |
| Unemployed | 36.2% | 39.5% | 17.0% |  |
| Other(student) | 4.2% | 3.7% | 7.5% |  |
| ***Living arrangements*** | **(n = 354)** | **(n = 301)** | **(n = 53)** |  |
| Home | 95.2% | 94.7% | 98.1% | 0.367 |
| Facility | 3.1% | 3.7% | 0.0% |  |
| Other | 1.7% | 1.7% | 1.7% |  |
| ***Cohabitants*** | **(n = 354)** | **(n = 301)** | **(n = 53)** |  |
| Living alone | 33.1% | 32.9% | 34.0% | 0.878 |
| Living with others | 66.9％ | 67.1% | 66.0% |  |
| ***Marital status*** | **(n = 352)** | **(n = 299)** | **(n = 53)** |  |
| Married | 48.6% | 46.5% | 60.4% | 0.358 |
| Never married | 25.0% | 25.8% | 20.8% |  |
| Divorced | 24.1% | 25.1% | 18.9% |  |
| Widowed | 2.0% | 2.3% | 0.0% |  |
| Not answered | 0.3% | 0.3% | 0.0% |  |
| ***Mental health and sleep quality*** |  |  |  |  |
| ***PSQI*** | **(n = 311)** | **(n = 301)** | **(n = 53)** |  |
| Good sleeper (≤5) | 25.7% | 23.7% | 36.7% | 0.055  (φ= 0.109) |
| Poor sleeper (>5) | 74.3% | 76.3% | 63.3% |  |
| ***PHQ-9*** | **(n = 338)** | **(n = 301)** | **(n = 53)** |  |
| screen-negative depression (<10) | 55.3% | 53.0% | 67.9% | <.05  (φ= 0.109) |
| screen-positive depression (≥10) | 44.7% | 47.0% | 32.1% |  |
| ***Previous treatment for AUD*** | **(n = 354)** | **(n = 301)** | **(n = 53)** |  |
| Yes | 51.4% | 55.5% | 28.3% | <.001  (φ = 0.194) |
| No | 48.6% | 44.5% | 71.7% |  |
| ***Drinking history*** | **Total** | **Inpatient** | **Outpatient** | ***p**** |
| ***Age at first alcohol use*** | **(n = 354)** | **(n = 301)** | **(n = 53)** |  |
| Mean ± SD  (min-max） | 17.7 ± 3.7 (1-30) | 17.9 ± 3.6  (3-30) | 16.5 ± 3.8  (1-21) | <.01  (φ = 0.683) |
| ***Age at onset of habitual drinking*** | **(n = 350)** | **(n = 301)** | **(n = 49)** |  |
| Mean ± SD  (min-max） | 23.7 ± 7.9 (13-64) | 24.1 ± 8.3  (13-64) | 21.8 ± 4.5  (16-40) | <.01 (φ =0.293) |
| ***Age at onset of drinking problems*** | **(n = 331)** | **(n = 283)** | **(n = 48)** |  |
| Mean ± SD  (min-max） | 40.3±13.6 (14-77) | 40.1±13.6  (14-77) | 41.6±13.5  (20-72) | 0.479 |
| ***Severity assessment by DSM-5*** | **Total** | **Inpatient** | **Outpatient** | ***p**** |
| Mild | 6.8% | 8.0% | 0.0% | 0.067  (φ = 0.124) |
| Moderate | 12.4% | 13.0% | 9.4% |  |
| Severe | 80.8% | 79.1% | 90.6% |  |

Note; Results are expressed as percentage or mean ±standard deviation and range.

AUD; alcohol use disorder

DSM5; Diagnostic and Statistical Manual of Mental Disorders, Fifth Edition; SCID-5-RV, Structured Clinical Interview for DSM-5 Research Version

**p* values for comparisons between inpatients and outpatients were calculated using the t-test or chi-square test.

**d* values and φ values indicate effect sizes.

**S5 Table:**

**Comparison of alcohol consumption patterns during the 90 days prior to baseline between inpatients and outpatients in the ALPS-J participants**

| **Average daily pure alcohol consumption (g)** | **Total**  **(n = 340)** | **Inpatient**  **(n = 292)** | **Outpatient**  **(n = 48)** | ***p****** | ***d****** |
| --- | --- | --- | --- | --- | --- |
| Mean ± SD  （min-max）  Median | 110.4 ± 70.6  (0.0-432.0)  100.0 | 112.00±72.72  (0.00-432.00)  100.0 | 100.36±56.00  (0.00-200.00)  108.0 | 0.291 | 0.165 |
| **Total amounts of pure alcohol consumption (g)** | **Total**  **(n = 339)** | **Inpatient**  **(n = 283)** | **Outpatient**  **(n = 56)** | ***p****** | ***d****** |
| Mean ± SD  （min-max）  Median | 8472.4 ± 6682.3  (0.0-38880.0)  7200.0 | 8545.29±6938.39  (0.00-38880.00)  7200.0 | 8019.66±4829.00  (0.00-18000.00)  7560.0 | 0.617 | 0.079 |
| **Number of heavy drinking days (days)** | **Total**  **(n = 304)** | **Inpatient**  **(n = 258)** | **Outpatient**  **(n = 46)** | ***p***** |  |
| Mean ± SD  （min-max）  Median | 65.5 ± 31.1 (1.0-90.0)  90.0 | 64.23±31.73  (1.00-90.00)  90.0 | 74.45±24.79  (13.00-90.00)  90.0 | 0.059 |  |

**Note**: All items of drinking status in the baseline survey were asked about participants' drinking habits over the 90 days before they entered the study, so the results in this table represent participants' drinking habits over the past 90 days before treatment began.

**p* values for comparisons between inpatients and outpatients were calculated using the t-test.

***p* values for comparisons between inpatients and outpatients were calculated using the Mann–Whitney U test.

**d* values indicate effect sizes.

Abbreviation: SD, Standard Deviation
